# Higher valent Pneumococcal Conjugate Vaccines: is it a roller coaster?

**DOI:** 10.3934/publichealth.2020004

**Published:** 2020-01-17

**Authors:** Godwin Oligbu

**Affiliations:** Paediatric Infectious Diseases Research Group, St. George's University of London, Cranmer Terrace, London SW17 0RE, UK

*Streptococcus pneumonia* (Pneumococcus, Spn), *Haemophilus influenzae* type b (Hib) and *Neisserria meningitidis* are encapsulated bacteria that can cause serious invasive infections, such as meningitis and septicaemia, in infants and young children. Their polysaccharide capsules are major virulence factors but also targets for protective antibody. In 1983, a 23-valent polysaccharide (PPV23) vaccine was licensed in the US, and included 11 additional serotypes to the 13-valent pneumococcal conjugate vaccine (PCV13), containing serotypes 2, 8, 9N, 10A, 11A, 12F, 15B, 17F, 20, 22F, and 33F. This vaccine (PPV23) however was associated with poor immunogenicity in young children below 18 months and the consequent inability of these infants to mount protective antibody responses against bacterial polysaccharide. This vaccine therefore failed to protect this vulnerable group of children from invasive bacterial diseases [1].

In order to overcome the poor immunogenicity of polysaccharide vaccines in infants, protein-polysaccharide conjugate vaccines were developed. Avery & Goebel in 1929 were the first to study the immunogenicity of polysaccharide antigens. They demonstrated that the poor immunogenicity of purified pneumococcal serotype 3 polysaccharide in mice could be enhanced by conjugation of the polysaccharide to a protein carrier [2]. This study and others was the genesis of the development of conjugate vaccines.

Unlike plain polysaccharide vaccine, the functional activity of antibodies elicited by conjugate vaccines, which is determined by its avidity, has been reported to be much higher [3]. This has been demonstrated to be directly associated with its complement-mediated bactericidal and opsonic activity and its ability to protect against bacteraemia in infant rats [3]. In addition, the high levels of antigen-specific antibodies induced by conjugate vaccines, have been shown to not only prevent invasive infections but also reduce asymptomatic nasopharyngeal carriage among vaccinated children [4], thereby reducing transmission to susceptible adults and unvaccinated children.

The above knowledge was used in the production of Hib conjugate vaccine in 1987, and became the first glycoconjugate vaccine licensed for use in the US infant routine immunization schedule in 1992. Shortly after, Meningococcal C (MenC) conjugate vaccine was introduced in 1999 in the UK and 2005 in the US. These vaccines led to a rapid decline in the incidence of both invasive Hib disease and MenC, with death following Hib rarely reported in the UK since 2011. The success of the Hib conjugate vaccines in reducing the incidence of invasive Hib disease in childhood has accelerated the development of conjugate vaccines designed to prevent infection by other encapsulated bacteria. It was vitally important that such vaccines formulation is able to render bacterial capsular polysaccharides immunogenic in those most at risk of infection.

In 2000, the 7-valent pneumococcal conjugate vaccine (PCV7) was licensed to protect against the seven most prevalent pneumococcal serotypes (4, 6B, 9V, 14, 18C, 19F and 23F) causing invasive pneumococcal disease (IPD) in children. Initially, in the US and at various times in other developed countries. In children, prior to the routine pneumococcal conjugate vaccination, the seven most common serotypes were responsible for 60–80% of all IPD in children [5]. This vaccine was effective and led to significant reduction in IPD caused by vaccine serotypes. However, the effect of this reduction was rapidly nullified by increase in IPD caused by non-vaccine serotypes. The main replacing serotypes varied in different countries but some of the emerging serotypes were consistently common, including serotypes 6C, 19A, 22F, 15 and 33 [6]. Replacement disease with serotype 19A was a particular problem in many parts of the world, but especially in the US, because this serotype was associated with resistance to multiple antibiotics [7].

In 2010–2011, PCV7 was replaced with one that protected against 13 serotypes (PCV13) in most industrialised countries and was aimed to protect against six additional serotypes (1, 3, 5, 6A, 7F and 19A). Another licensed PCV formulation; 10-valent pneumococcal conjugate vaccine (PCV10) that protects against serotypes 1, 5 and 7F in addition to PCV7 serotypes was also licensed at around the same time and implemented in other countries. These vaccines have led to rapid and sustained declines in IPD, including meningitis caused by the respective vaccine serotypes, in both healthy children and in those with comorbidities [8],[9]. Widespread availability of PCVs has reduced the burden of IPD substantially in children under 5 years of age, from over 800,000 annual deaths before PCV introduction to 541,000 deaths in 2008 [10]. However, with all the PCVs, the overall reduction in IPD has been offset by an increase in IPD caused by non-vaccine serotypes (NVT) [11]. Currently, nearly all pneumococcal infections in children are caused by non-vaccine pneumococcal serotypes.

The most common serotypes now causing IPD in Europe include 8, 3, 22F, 12F, 19A, 9N, 7F, 15A, 33F, 10A, accounting for 62% of typed isolates. Of the cases in children under 5 years of age, 72% were caused by a serotype not included in any PCV. Among cases aged 65 years and over, 71% were caused by a PPV23 serotype, and 32% were caused by a PCV13 serotype especially in the elderly [12].

Since these serotypes have only emerged after the pneumococcal vaccines were introduced, there is very little knowledge of the risk, clinical severity and outcomes of IPD caused by these new and emerging serotypes. While recent studies have focus on the clinical virulence of the replacing serotypes, there is need to curb these replacing serotypes with a PCV that is capable of providing a wider coverage for the major serotypes now responsible for pneumococcal diseases worldwide. Recently, 15-valent pneumococcal conjugate vaccine (PCV15) was announced in the US and contain 2 additional serotypes, 22F and 33F to PCV13. This vaccine has demonstrated adequate immune response to all the serotypes in PCV13 and the additional 2 serotypes, 22F and 33F, both in healthy infants and adults [13],[14]. In a recent longitudinal observational study of children with IPD in the UK, who are at higher risk of IPD, demonstrated that serotypes 23B, 24F, 15B/C, 22F and 12F formed majority serotypes causing IPD [15]. In addition, children with sickle cell disease (SCD) have been shown to be particularly at increase risk of serogroup 15, with majority of IPD in children with SCD now due to serotype 15B/C. This children are therefore not likely to be adequately covered by PCV15 and would continue to require PPV23 prophylaxis [16],[17]. Another higher conjugate vaccine, 20-valent pneumococcal conjugate vaccine (PCV20), currently in the very early phase of clinical trial and contains serotypes 8, 10A, 11A, 12F, 15B, 22F, and 33F, in addition to PCV13 serotypes, have been found to display acceptable safety profiles and induce serotype-specific immune responses comparable to PCV13 [18]. The PCV15 are likely to reduce a significant percentage of the replacing serotypes, by an additional 10 to 15% in one US adult study, including children who are at higher risk of IPD, who are more likely to suffer from significant morbidity and mortality. It is more likely that a more significant proportion of the replacing serotypes will be reduced by PCV20 if licensed. However, given our experience with PCV7 and PCV13, It is also likely that this higher valent conjugate vaccines will create a niche in the current nasopharyngeal carriage and result into further serotype replacement. It is therefore most likely that as PCV is extensively used, the pneumococci will continue to adapt to vaccine pressure. Although some anecdotal evidence has shown that these replacing serotypes are likely to be less invasive while some have suggested that these niche might be replaced by *staphylococcus aureus*. Nonetheless, while we welcome this novel vaccines to reduce the current burden of IPD, it is pertinent that a serotype independent vaccine or whole cell vaccine would be required to curb this replacing serotypes in the future [19].
